# Acknowledgment to reviewers—November 2020 to October 2021

**DOI:** 10.1080/20961790.2021.2017608

**Published:** 2022-01-28

The Editors of the *Forensic Sciences Research* (*FSR*) wish to thank the following people, and any other reviewer whose name has been inadvertently omitted, for giving your time and expertise to review papers and facilitate the smooth running of *FSR* between November 2020 and October 2021.

Augustine Kolapo Ademola, *Nigeria*

Miriam Baeta, *Spain*

Eric J. Bartelink, *USA*

Susanne Bengtson, *Denmark*

John William Berketa, *Australia*

Carol Berkowitz, *USA*

Carla Bini, *Italy*

Alessandro Bonsignore, *Italy*

Nathan W. Bower, *USA*

Michael P. Caligiuri, *USA*

Alessandra Caso, *UK*

Valerio Causin, *Italy*

Markéta Čechová, *Czech Republic*

Xiaohong Chen, *China*

Derek Congram, *Canada*

Diana Dias da Silva, *Portugal*

Shanlin Fu, *Australia*

Binita Gandhi, *India*

Yuzhen Gao, *China*

Dominic Gascho, *Switzerland*

Lorenzo Gitto, *USA*

Tony Godet, Switzerland

Michael S. Gordon, *Australia*

Fei Guo, *China*

Rafizah Mohd Hanifa, *Malaysia*

Bryan T. Johnson, *USA*

Kaveh Jorabchi, *USA*

Kelly Kamnikar, *USA*

Shakeel Kazmi, *Pakistan*

Michael Kennedy, *Australia*

Lay See Khoo, *Malawi*

Ardavan Khoshnood, *Sweden*

Raj Kumar, *India*

Zhengdong Li, *China*

Shiquan Liu, *China*

Ningguo Liu, *China*

Helen M. Liversidge, *UK*

Joaquin Lucena, *Spain*

Yehui Lvy, *China*

Dong Ma, *China*

Áurea Madureira-Carvalho, *Portugal*

Edgard Michel-Crosato, *Brazil*

Ruben Miranda, *Spain*

Molly Miranker, *USA*

Linton A. Mohammed, *USA*

David Senhora Navega, *Portugal*

Zuzana Obertová, *Australia*

José Restolho, *Portugal*

Rachel Lima Ribeiro Tinoco, *Brazil*

Servaas Rossouw South, *Africa*

Calin Scripcaru, *Romania*

Muhammad Shahzad, *Pakistan*

Yu Shao, *China*

M. Paz Suarez-Mier, *Spain*

Qiran Sun, *China*

Susan Vanderplas, *USA*

Milena Vásquez-Amézquita, *Colombia*

Fang Wang, *China*

Qi Wang, *China*

Zheng Wang, *China*

Linzi Wilson-Wilde, *Australia*

Hui Yan, *China*

Shaohua Yi, *China*

Rabaa Zaibi, *Tunisia*

Maoxiu Zhang, *USA*

Cuiling Zhang, *China*

Qinting Zhang, *China*

Suhua Zhang, *China*

Xinqing Zhang, *China*

